# Synthetic Patient–Physician Conversations Simulated by Large Language Models: A Multi-Dimensional Evaluation

**DOI:** 10.3390/s25144305

**Published:** 2025-07-10

**Authors:** Syed Ali Haider, Srinivasagam Prabha, Cesar Abraham Gomez-Cabello, Sahar Borna, Ariana Genovese, Maissa Trabilsy, Bernardo G. Collaco, Nadia G. Wood, Sanjay Bagaria, Cui Tao, Antonio Jorge Forte

**Affiliations:** 1 Division of Plastic Surgery, Mayo Clinic, Jacksonville, FL 32224, USA; 2 Department of Radiology AI IT, Mayo Clinic, Rochester, MN 55905, USA; 3 Division of Surgical Oncology, Mayo Clinic, Jacksonville, FL 32224, USA; 4 Department of Artificial Intelligence and Informatics, Mayo Clinic, Jacksonville, FL 32224, USA; 5 Center for Digital Health, Mayo Clinic, Rochester, MN 55905, USA

**Keywords:** synthetic data, medical consultations, large language models, medical simulation, patient–physician interaction, clinical communication

## Abstract

Background: Data accessibility remains a significant barrier in healthcare AI due to privacy constraints and logistical challenges. Synthetic data, which mimics real patient information while remaining both realistic and non-identifiable, offers a promising solution. Large Language Models (LLMs) create new opportunities to generate high-fidelity clinical conversations between patients and physicians. However, the value of this synthetic data depends on careful evaluation of its realism, accuracy, and practical relevance. Objective: To assess the performance of four leading LLMs: ChatGPT 4.5, ChatGPT 4o, Claude 3.7 Sonnet, and Gemini Pro 2.5 in generating synthetic transcripts of patient–physician interactions in plastic surgery scenarios. Methods: Each model generated transcripts for ten plastic surgery scenarios. Transcripts were independently evaluated by three clinically trained raters using a seven-criterion rubric: Medical Accuracy, Realism, Persona Consistency, Fidelity, Empathy, Relevancy, and Usability. Raters were blinded to the model identity to reduce bias. Each was rated on a 5-point Likert scale, yielding 840 total evaluations. Descriptive statistics were computed, and a two-way repeated measures ANOVA was used to test for differences across models and metrics. In addition, transcripts were analyzed using automated linguistic and content-based metrics. Results: All models achieved strong performance, with mean ratings exceeding 4.5 across all criteria. Gemini 2.5 Pro received mean scores (5.00 ± 0.00) in Medical Accuracy, Realism, Persona Consistency, Relevancy, and Usability. Claude 3.7 Sonnet matched the scores in Persona Consistency and Relevancy and led in Empathy (4.96 ± 0.18). ChatGPT 4.5 also achieved perfect scores in Relevancy, with high scores in Empathy (4.93 ± 0.25) and Usability (4.96 ± 0.18). ChatGPT 4o demonstrated consistently strong but slightly lower performance across most dimensions. ANOVA revealed no statistically significant differences across models (F(3, 6) = 0.85, *p* = 0.52). Automated analysis showed substantial variation in transcript length, style, and content richness: Gemini 2.5 Pro generated the longest and most emotionally expressive dialogues, while ChatGPT 4o produced the shortest and most concise outputs. Conclusions: Leading LLMs can generate medically accurate, emotionally appropriate synthetic dialogues suitable for educational and research use. Despite high performance, demographic homogeneity in generated patients highlights the need for improved diversity and bias mitigation in model outputs. These findings support the cautious, context-aware integration of LLM-generated dialogues into medical training, simulation, and research.

## 1. Introduction

Simulation plays a crucial role in medical education, providing a safe and controlled environment for learners to practice clinical skills and develop decision-making abilities [1,2]. From clinical vignettes to standardized patient interactions during Objective Structured Clinical Examinations (OSCEs), simulation has long been foundational in training competent and compassionate healthcare providers [3,4]. These methods not only test clinical knowledge but also assess interpersonal communication and professionalism, which are key competencies in patient-centered care.

In parallel, simulation has emerged as a crucial tool in the development of artificial intelligence (AI) systems for healthcare [5,6]. Just as patient actors bring clinical scenarios to life for medical trainees, synthetic data can power the training and evaluation of AI models, particularly in medical dialogue systems (MDS) and healthcare chatbots [4,7,8].

Synthetic data is artificially generated information that mirrors the statistical properties of real datasets without containing actual individual data [9]. Unlike anonymized data, it has no traceable identifiers. Techniques such as Generative Adversarial Networks (GANs) and Variational Autoencoders (VAEs) are prominent examples [10,11], capable of learning complex data distributions to produce realistic synthetic samples across diverse modalities [9,12,13]. Large Language Models (LLMs) are uniquely positioned to generate high-fidelity, realistic, and privacy-preserving conversational data [14].

In recent years, simulation has taken on a new dimension with the emergence of large language models (LLMs), which can generate synthetic clinical data, most notably, artificial patient–physician conversations [15,16,17]. While patient–physician interactions are increasingly being recorded using ambient sensing tools such as Abridge, access to the resulting transcripts remains tightly restricted due to privacy concerns and the need to protect sensitive patient information [18]. Medical records, including transcripts of patient–physician interactions, contain personal health information that is protected under regulations such as the Health Insurance Portability and Accountability Act (HIPAA) in the United States [19]. These privacy regulations create three primary constraints for AI development: data scarcity due to restricted access to real patient conversations, ethical barriers regarding patient consent and potential misuse of sensitive health information, and logistical challenges in obtaining, de-identifying, and processing clinical dialogue data at scale for research and training purposes.

The generation of synthetic medical dialogues using LLMs offers a compelling alternative, bypassing these constraints while enabling large-scale, controlled experimentation, which opens up new possibilities for research, education, and AI development [20]. These models can engage in role-play, simulating either patients or physicians, and produce conversations that mimic the structure, tone, and content of real clinical interactions, without containing any protected health information. Moreover, synthetic dialogues can be tailored to cover a wide array of clinical scenarios, allowing for rigorous and scalable testing of chatbots and MDS, without compromising patient safety or privacy [21].

Plastic surgery presents a particularly challenging and instructive test case for evaluating synthetic medical dialogues. It not only requires precision in medical knowledge and procedural explanation but also demands emotional intelligence and empathetic communication around body image, aesthetic goals, and psychological well-being [22]. Conversations in this context are nuanced, personal, and often complex in terms of ethics. Comprehensive plastic surgery consultations involve multiple essential components, including medical history assessment, patient goal exploration, detailed procedure explanation, risk-benefit discussion, informed consent processes, post-operative care planning, and addressing psychological considerations around body image and aesthetic expectations. These consultations require physicians to seamlessly integrate technical medical knowledge with empathetic communication, making them ideal scenarios for testing LLM capabilities in generating complex, multi-dimensional clinical interactions. These features make plastic surgery an ideal domain for assessing whether LLMs can generate consultation transcripts that are not only accurate but also realistic, human-centered, and educationally useful [23].

Although prior work has explored synthetic dialogue generation from clinical notes [24] or structured inputs, no studies, to the best of our knowledge, have directly evaluated the performance of LLMs using zero-shot prompt input, combined with expert review. There is a need for more holistic, expert-guided assessments of synthetic dialogues.

The objective of this study was to quantitatively and qualitatively assess the performance of four leading LLMs: ChatGPT 4.5, ChatGPT 4o, Claude 3.7 Sonnet, and Google Gemini Pro 2.5 in generating synthetic patient–physician interactions for common plastic surgery consultation scenarios. These transcripts were produced using standardized prompts and evaluated by clinically trained experts across seven dimensions, including medical accuracy, realism, empathy, and educational usability, as well as through quantitative transcript analysis of aspects such as lexical diversity, physician-to-patient dialogue ratio, readability, etc. In doing so, this study aims to provide an evidence-based evaluation of LLMs’ capabilities in clinical communication tasks and to explore their potential role in simulation, education, and research, particularly where real conversational data remains inaccessible or ethically sensitive. As these ambient intelligence sensors evolve, integrating data from microphones, cameras, and wearable biosensors into simulation pipelines presents an opportunity to generate or validate synthetic dialogues that include not only verbal content but also non-verbal and physiological cues in Virtual Reality (VR) environments [25]. This paper will subsequently elaborate upon broader ethical considerations pertaining to fairness, transparency, and safety inherent in the generation of synthetic dialogues.

## 2. Methods

### 2.1. Study Design

This prospective, comparative study evaluated the quality of synthetic patient–physician dialogues generated by four large language models (LLMs): ChatGPT 4.5 [26] (OpenAI—San Francisco, CA, USA), ChatGPT 4o [27] (OpenAI—San Francisco, CA, USA), Claude 3.7 Sonnet [28] (Anthropic—San Francisco, CA, USA), and Google Gemini Pro 2.5 [29] (Google—Mountain View, CA, USA). These four models were selected based on their status as leading general-purpose LLMs with demonstrated strong performance across diverse natural language tasks. ChatGPT 4.5 and 4o represent OpenAI’s latest iterations, with 4o offering enhanced multimodal capabilities and improved reasoning. Claude 3.7 Sonnet is Anthropic’s most recent model, known for nuanced conversational abilities and safety-focused training. Google Gemini Pro 2.5 represents Google’s advanced multimodal AI system with impressive performance in complex reasoning tasks. While none of these models were specifically fine-tuned for medical applications, they were chosen as representatives of current state-of-the-art general-purpose LLMs to evaluate baseline capabilities in medical dialogue generation without domain-specific training. Ethical approval was not required as no human subjects were directly involved in data generation.

### 2.2. Transcript Generation

A standardized prompt, varied only by surgery name (abdominoplasty, blepharoplasty, facelift, hand surgery, lymphedema surgery, breast reconstruction, rhinoplasty, breast augmentation, liposuction, and mastopexy), was used for ten clinical scenarios. The prompt directed LLMs to generate realistic pre- and post-operative patient–physician dialogues, including natural speech, emotional nuance, shared decision-making, and relevant clinical/lifestyle details. The exact prompt used across all models was as follows:

“Simulate a realistic, synthetic transcript that captures pre-operative and post-operative consultations between a physician and a patient undergoing [Procedure]. The dialogue should reflect a dynamic and authentic medical interaction, incorporating natural speech patterns, emotional nuance, and interpersonal rapport. It should feature a detailed patient demographic profile, including lifestyle factors and relevant social or environmental stressors that may influence care. The transcript must include shared decision-making moments, where the physician explains options and the patient engages with questions or concerns. Where appropriate, include objective clinical data to enhance the medical realism of the exchange.”

This zero-shot prompting approach evaluated each model’s inherent dialogue generation capabilities without providing example conversations or task-specific training. No model-specific prompt optimization was performed to ensure fair comparative evaluation. This approach ensured consistency for fair model comparison by testing LLMs on their variability in interpreting prompts for dialogue generation. In total, 40 unique synthetic patient–physician dialogue transcripts (4 LLMs × 10 scenarios) were generated, each representing a complete, simulated pre- and post-operative consultation. The mean transcript generation time was 23.6 s. LLM platform access requires monthly subscriptions totaling approximately $57 across the four models.

### 2.3. Evaluation Framework and Process

**Figure 1:**
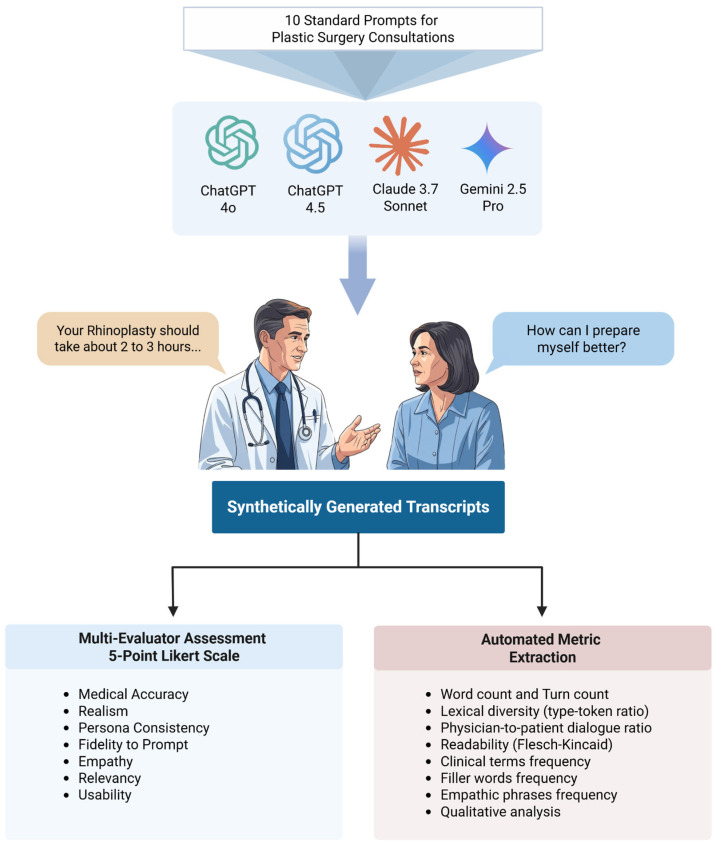
Study Workflow Overview: 40 transcripts in total (10 scenarios × 4 LLMs).

A standardized rubric with seven criteria, informed by prior literature on synthetic dialogue evaluation (Gray et al., Cook et al., Das et al., Moser et al.), was developed to assess dialogue quality. The complete evaluation rubric with specific performance indicators for each score (1–5) across all seven criteria is provided in Supplementary Table S1. Three clinically trained researchers (MD/MBBS) from the plastic surgery department independently evaluated all 40 transcripts (Figure 1).

The rubric, developed with insights from Gray [30], Cook [15], Das [24], and Moser [31], prioritized realism and authenticity, fidelity and factual consistency, and a balance of linguistic quality with informational accuracy. Each criterion was rated on a 5-point Likert scale (1 = Very Poor to 5 = Excellent):

1. Medical Accuracy: Correctness and appropriateness of medical information, advice, and terminology used within the dialogue.
2. Realism: Plausibility and authenticity of the conversation, reflecting how a real patient–physician interaction might unfold.
3. Persona Consistency: Maintenance of consistent patient and physician personas (e.g., personality, knowledge level, emotional state) throughout the dialogue.
4. Fidelity to Prompt: Adherence of the generated dialogue to the specific details, constraints, and objectives outlined in the input scenario prompt.
5. Empathy: Demonstration of understanding, compassion, and appropriate emotional responsiveness by the physician persona towards the patient persona.
6. Relevancy: Focus of the dialogue on the core clinical issue presented in the scenario, avoiding irrelevant tangents or information.
7. Usability (for training/simulation): The overall suitability and potential effectiveness of the dialogue for use in medical education, training, or simulation exercises.

Before formal evaluation, evaluators calibrated their scoring using three pilot transcripts (not part of the main study). They independently scored these, then discussed as a group to clarify interpretations and ensure a consistent approach.

### 2.4. Quantitative Transcript Analysis and Metric Extraction

A custom Python (v3.13) script in PyCharm (v2024.3.5) was used to analyze all transcripts for textual metrics: word count, turn count, lexical diversity (which measures the variety of words used in a text, typically calculated as the type-token ratio), physician-to-patient dialogue ratio, and readability (Flesch Reading Ease, Flesch-Kincaid Grade Level). The type-token ratio was calculated as the number of unique words (types) divided by the total word count (tokens), where higher ratios indicate greater lexical diversity and vocabulary richness. For example, in a phrase containing 10 total words (tokens) with 7 unique words (types), the type-token ratio would be 0.7, indicating moderate lexical diversity. Domain-specific clinical content was quantified using a custom plastic surgery lexicon. Additional metrics included filler word frequency (to assess conversational realism) and empathic phrase frequency (based on a manually curated list). Transcripts from each model were then consolidated for further qualitative analysis.

### 2.5. Statistical Analysis

SPSS 29.0.2.0 was used for statistical analysis. Descriptive statistics (mean, SD, median) were calculated for seven evaluation criteria across four LLMs. A two-way repeated measures ANOVA assessed overall dialogue quality, with ‘LLM Model’ and ‘Evaluation Metric’ as within-subjects factors and Likert score as the dependent variable. For quantitative transcript analysis, mean scores for each metric were computed per model.

## 3. Results

### 3.1. Descriptive Statistics

**Figure 2:**
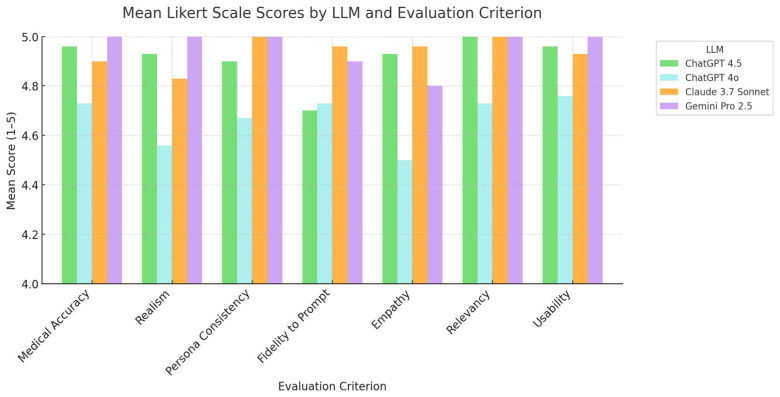
Bar graphs comparing Mean Likert scale scores for each evaluation criterion across four large language models (LLMs) in generating synthetic plastic surgery transcripts.

All four LLMs generated high-quality synthetic dialogues, with mean scores generally above 4.5 on a 5-point Likert scale for most criteria, indicating ‘Good’ to ‘Excellent’ quality as perceived by clinical evaluators. Detailed scores are in Table 1, with a comparative visualization in Figure 2.

Google Gemini Pro 2.5 achieved perfect mean scores (5.00 ± 0.00) for Medical Accuracy, Realism, Persona Consistency, Relevancy, and Usability. Claude 3.7 Sonnet also scored perfectly for Persona Consistency and Relevancy and rated highly on other criteria like Empathy (4.96 ± 0.18). ChatGPT 4.5 showed strong, consistent performance, with a perfect score for Relevancy (5.00 ± 0.00) and near-maximum scores in most other areas. ChatGPT 4o generally had slightly lower, though still strong (all > 4.5), ratings, particularly in Empathy (4.50 ± 0.73) and Realism (4.56 ± 0.56). The perfect mean scores for Gemini Pro 2.5 and Claude 3.7 Sonnet on several criteria indicate unanimous evaluator agreement on their high performance in those aspects.

A two-way repeated measures ANOVA (Model x Evaluation Metric) revealed no statistically significant main effect for Model (F(3, 6) = 0.85, *p* = 0.52, Greenhouse-Geisser corrected *p* = 0.46) or Evaluation Metric (F(6, 12) = 1.39, *p* = 0.29, corrected *p* = 0.35). The Model x Metric interaction was also non-significant (F(18, 36) = 1.18, *p* = 0.33, corrected *p* = 0.41). Thus, despite descriptive score variations, no significant differences were found across models, metrics, or their interaction.

### 3.2. Transcript Content Analysis

**Figure 3:**
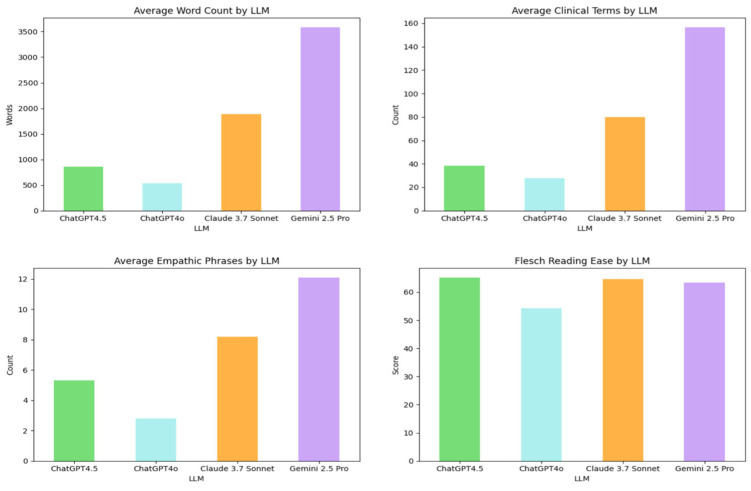
Bar charts comparing linguistic features across four LLMs.

Quantitative analysis revealed substantial variations in conversational characteristics across the LLMs, as detailed in Table 2. (Figure 3 may offer a visual comparison of these metrics.)

**Figure 4:**
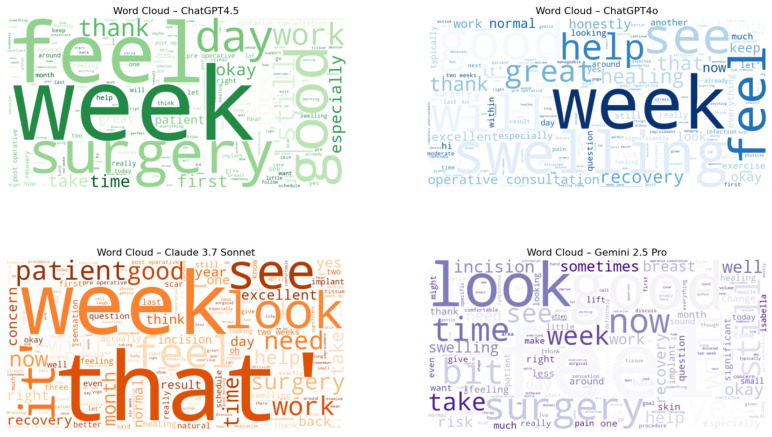
Word Clouds of Most Frequent Terms by LLM.

Gemini 2.5 Pro and Claude 3.7 Sonnet produced markedly longer dialogues with higher turn counts, whereas ChatGPT4o generated the briefest and most concise exchanges (Table 2). The physician-to-patient word ratio indicated varying degrees of physician speech dominance, with ChatGPT4o and Gemini 2.5 Pro showing a higher ratio (Table 2). Lexical diversity was highest in ChatGPT4o outputs and lowest in those from Gemini 2.5 Pro. Word clouds visualizing the most frequent terms by LLM are presented in Figure 4.

Regarding content and nuance, Gemini 2.5 Pro dialogues contained the highest mean number of clinical terms, filler words, and empathic phrases. A custom library was created containing empathetic phrases such as ‘I understand,’ ‘I am sorry,’ ‘that’s completely normal,’ ‘thank you for sharing,’ and ‘you’re not alone.’ Similarly, a filler word library included terms like ‘um,’ ‘well,’ ‘you know,’ ‘I mean,’ and ‘actually’ to assess conversational realism. Conversely, ChatGPT4o used these elements least frequently (Table 2). Readability scores (Flesch Reading Ease and Flesch-Kincaid Grade Level) indicated that all outputs were generally accessible to lay audiences, with some variations as noted in Table 2.

These findings underscore key stylistic and structural differences. While all models produced medically plausible dialogues, their output varied considerably in length, verbosity, lexical richness, clinical density, and affective tone (Table 2), influencing their potential suitability for diverse applications like simulation, patient education, or healthcare training.

### 3.3. Patient and Clinical Profile Characteristics

Synthetic transcript analysis revealed patterns in demographic and clinical detail generation. All 40 patient profiles were female, indicating a significant gender imbalance. Physician demographics were more varied: of 42 identified, 52.4% (n = 22) were female, 14.3% (n = 6) male, and 33.3% (n = 14) had unspecified gender.

Patient age distribution was limited: 55% were 25–44 years and 45% were 45–65 years, with no representation from 0 to 18, 19–24, or 65+ age groups, reflecting a lack of age diversity. Name repetition was also noted (e.g., Maria [10 times], Sarah [7], Isabella [3]; others included Linda, Julia, Carla, and Eleanor), suggesting restricted name generation variability.

Dialogues often focused on common medical conditions: hypertension and diabetes (8 profiles each) were most frequent, followed by seasonal allergies (7), asthma (6), and arthritis (4). Allergy references included pollen/ragweed (7 instances) and penicillin (3), likely reflecting patterns from training data.

Medication references also reflected standard practices. Antibiotics were most common (24 patients), followed by prescription pain medications (17), silicone-based scar treatments and NSAIDs (13 each), Lisinopril (11), Tylenol (10), and Albuterol (4). These patterns suggest a preference for widely recognized treatments.

## 4. Discussion

This study evaluated four leading LLMs (ChatGPT 4.5, ChatGPT 4o, Claude 3.7 Sonnet, and Google Gemini Pro 2.5) for generating synthetic patient–physician dialogues in plastic surgery. Core findings indicate high proficiency across all models, with mean scores generally exceeding 4.5 (out of 5) for medical accuracy, realism, persona consistency, prompt fidelity, empathy, relevancy, and usability.

Descriptively, Google Gemini Pro 2.5 and Claude 3.7 Sonnet frequently achieved perfect or near-perfect mean scores, suggesting outstanding quality according to clinical evaluators. However, a two-way repeated measures ANOVA found no statistically significant differences in rated dialogue quality based on the LLM, evaluation metric, or their interaction. This suggests leading LLMs have reached a high baseline quality for generating such conversations, possibly indicating a ceiling effect with the 5-point Likert scale that may obscure differentiation among excellent models.

Beyond subjective ratings, quantitative transcript analysis revealed notable stylistic and structural variations. Gemini 2.5 Pro produced the longest, most verbose dialogues with the highest density of clinical terms and empathic phrases. In contrast, ChatGPT4o generated the most concise outputs. While all models were generally readable, these differences in length, lexical diversity, and physician-to-patient speech ratio highlight distinct conversational profiles despite similar quality ratings. Such variations could influence their suitability for specific uses, like detailed educational simulations versus brief, focused interactions.

### 4.1. The Translational Impact of LLM-Generated Synthetic Data in Healthcare

The critical need for accessible, high-quality patient data in healthcare, often impeded by stringent privacy regulations, has propelled synthetic data to the forefront of innovation [32].

Synthetic data in healthcare circumvents challenges like the scarcity of diverse datasets (especially for rare diseases or underrepresented groups), ethical and logistical complexities of real data collection, and the costs of accessing sensitive patient information [33]. LLM-generated synthetic dialogues offer a scalable, customizable, and de-identified alternative, unlocking new avenues for research, education, and AI development while ensuring privacy and regulatory compliance [34].

These findings significantly impact medical simulation and education. Simulation is foundational in healthcare training, offering a safe environment for skill practice. Our results support LLMs’ ability to generate high-quality, realistic patient–physician dialogues as advanced Virtual Standardized Patients (VSPs) [7]. These synthetic interactions allow learners to practice complex competencies like history-taking, shared decision-making, and empathetic communication, especially for rare, sensitive, or logistically challenging scenarios. The LLMs’ demonstrated persona consistency and empathy, reflected in high evaluation scores, enhance the educational fidelity of these encounters.

Furthermore, LLM-generated dialogues are promising for developing and evaluating Medical Dialogue Systems (MDS) and conversational AI [24]. The scarcity of privacy-compliant, realistic conversational data hinders training and testing these AI tools. Our approach provides a scalable, ethical data source for training conversational AI and evaluating other clinical LLMs, akin to sophisticated frameworks like CRAFT-MD [35]. The consistently high scores for ‘Medical Accuracy,’ ‘Realism,’ and ‘Empathy’ in our synthetic plastic surgery dialogues affirm LLMs’ capacity to navigate the medical knowledge and emotional dynamics of these consultations.

### 4.2. Relationship to Real-World Patient–Physician Conversations

Real-world primary care conversations in the U.S. average around 11–18 min [36,37], while Emergency Department encounters involve approximately 19 min of direct provider conversation [38]. Optimal speech rates for comprehension range from 120 to 200 words per minute [39]. Based on this, dialogues from Gemini 2.5 Pro (mean ~3700 words) and Claude 3.7 Sonnet (mean ~1900 words) translate to approximately 30 and 16 min of spoken interaction, respectively. These durations often exceed clinical averages, suggesting their verbosity, while beneficial for detailed educational simulations, may not fully align with typical conversational patterns. Future work could explore prompting for more concise dialogues that better reflect clinical time constraints.

Physicians often dominate talk time in real-world consultations (physician-to-patient ratios ~1.13–1.45) [40] and ask most questions [41]. Our LLM-generated dialogues, with ratios from 1.3 (ChatGPT 4.5, Claude 3.7 Sonnet) to 1.7 (ChatGPT 4o, Gemini 2.5 Pro), show varying physician speech dominance generally aligning with these real-world variations. However, while these synthetic conversations scored high in ‘Realism’ for their fluid and effective communication flows, they did not depict the frequent interruptions patients experience in actual encounters [42].

Physician empathy is strongly linked to improved patient outcomes [43]. Evaluators rated LLM dialogues highly for ‘Empathy’ (e.g., Claude 3.7 Sonnet at 4.96; ChatGPT 4.5 at 4.93), with Gemini 2.5 Pro generating the most empathic phrases. The consistently high scores suggest that the LLMs’ empathetic expressions are perceived as authentic in the given scenarios.

Optimal healthcare communication is patient-centered, with minimal interruptions and genuine empathy. The high scores for our synthetic dialogues in ‘Medical Accuracy,’ ‘Realism,’ and ‘Usability’ (e.g., Gemini 2.5 Pro at 5.00 for all) underscore their potential to foster these qualities in education and research. By providing accurate, realistic, and communicatively effective simulations, these LLM-generated conversations are invaluable tools for training healthcare professionals. The ability of these leading LLMs to emulate such complex interactions with high fidelity, validated by medically trained reviewers, is a significant step in addressing data gaps in medical education and AI development.

### 4.3. Challenges and Limitations of LLM-Generated Synthetic Patient–Physician Conversations

While demonstrating LLMs’ capabilities, this study also highlights inherent challenges. Accuracy and reliability are key concerns since LLMs can “hallucinate” inaccurate information [44]. Though our evaluators noted high ‘Medical Accuracy,’ this risk demands vigilance, especially for clinical decision-making applications.

Bias propagation, observed in our findings, represents a significant limitation with broader implications for AI-generated healthcare content. The models generated 100% female patient profiles across all 40 scenarios, despite no gender specification in our prompts, indicating systematic bias likely stemming from training data that disproportionately represents women in plastic surgery contexts. This bias likely reflects historical patterns in medical literature, media coverage, and online discussions where plastic surgery has been predominantly associated with female patients, despite significant male participation in both cosmetic and reconstructive procedures.

The age concentration only in the 25–44 (55%) and 45–65 (45%) age ranges, completely excluding pediatric and older adult demographics, may reflect a training data bias toward the most commonly discussed age groups for elective cosmetic procedures. LLMs tend to generate content based on the most frequent patterns in their training data, which may overrepresent certain demographics while underrepresenting others, particularly vulnerable populations like children requiring reconstructive surgery or elderly patients seeking cosmetic procedures.

The repetitive name generation (e.g., “Maria” appearing 10 times) suggests that the models may be drawing from limited, culturally biased datasets or defaulting to the most statistically common names in their training corpora, rather than generating truly diverse patient representations. This pattern indicates that LLMs may perpetuate not only gender and age biases, but also cultural and ethnic biases present in their training data.

These systematic biases pose serious concerns for educational and research applications. Training medical students or AI systems on such non-representative synthetic data could inadequately prepare healthcare providers for diverse patient populations, potentially contributing to care disparities. The exclusive focus on female patients could reinforce misconceptions about who seeks plastic surgery, while the age limitations could leave trainees unprepared for pediatric or geriatric cases. Such biases highlight the critical need for bias mitigation strategies, inclusive prompt engineering approaches, and careful oversight when deploying LLM-generated content in medical education and AI development [45].

Synthetic data generation also introduces the risk of noise [46], including medical inaccuracies, inconsistent personas, unnatural conversational patterns, and repetitive language structures that could compromise educational utility. Comprehensive noise mitigation in synthetic healthcare data requires ongoing vigilance, including multi-stage quality filtering, ensemble generation [47] using multiple models with consensus-based selection, domain-specific fine-tuning to reduce medical inaccuracies [48], bias detection algorithms, and iterative refinement processes to ensure the reliability and safety of AI-generated medical content for educational and research applications.

Privacy is another hurdle, as most publicly available LLMs are not inherently HIPAA-compliant [49]. While our study used non-identifiable scenarios, broader applications require robust de-identification to prevent re-identification from source data if real patient records are involved.

Ensuring complete realism, despite high ‘Realism’ scores, presents ongoing issues. Synthetic dialogues may not fully capture subtle language, natural disfluencies, or precise emotional nuances. Our analysis indicated LLM conversations could be overly verbose or use atypical vocabulary. The preference for common conditions (e.g., Hypertension or Diabetes) and medications also limits scenario diversity, potentially overlooking rare or atypical presentations, and handling multi-turn conversational complexity remains challenging.

Further limitations include the absolute necessity for expert medical review of generated content, which may potentially reduce time savings. The absence of non-verbal cues in text-based dialogues creates a significant fidelity gap. Finally, the evolving LLM landscape requires clearer regulatory certainty for synthetic data in healthcare, which is further complicated by the scarcity of high-quality, privacy-compliant, and conversational real-world datasets for training truly tailored medical LLMs.

### 4.4. Strengths of the Study

This study is the first comparative evaluation of leading pre-trained LLMs for synthetic clinical dialogue generation, establishing a benchmark for AI-generated medical conversations. Our head-to-head comparison of four state-of-the-art models using standardized prompts revealed their distinct performance capabilities.

A total of 3 clinically trained evaluators independently assessed all transcripts in a blinded design after formal calibration, yielding 840 total evaluations (10 transcripts × 7 criteria × 3 evaluators × 4 LLMs). This expert clinical assessment was uniquely paired with novel automated content analysis of linguistic properties, conversational dynamics, and clinical terminology, providing both subjective expert validation and objective quantitative benchmarking not previously achieved.

The choice of plastic surgery, demanding integration of technical medical knowledge with empathetic communication on sensitive topics, served as a challenging test for zero-shot dialogue generation and an ideal proving ground for LLM capabilities in complex interactions without structured input.

By assessing training utility and benchmarking against authentic clinical conversation characteristics, this work offers immediate practical guidance for medical educators and establishes a replicable evaluation framework, accelerating the responsible integration of AI-generated content in medical training.

### 4.5. Limitations of the Study

Several limitations warrant consideration. Our intentional focus on plastic surgery, while a rigorous test case, limits direct generalizability to other medical domains. The evaluation of 10 scenarios by 3 experts, though sufficient for our comparison, is a focused assessment that could be expanded with broader clinical perspectives and larger samples. Methodologically, the 5-point Likert scale may have limited ability to detect subtle differences between high-performing models, as indicated by multiple perfect scores. Our standardized prompt strategy, while ensuring control, is one approach; varied prompting techniques might reveal further model capabilities. The evaluation framework involved evaluators from a single institution and specialty. Although calibration was performed, future evaluations would benefit from diverse perspectives (other specialties, institutions, patients, and educators). Evaluators were also aware of the synthetic nature of the dialogues. Our analysis centered on expert evaluation and automated content analysis. We did not directly compare synthetic dialogues with authentic transcripts from identical scenarios, nor did we assess real-world educational effectiveness or learning outcomes, which are important next steps for validating the practical utility. Finally, this evaluation is a snapshot from May 2025. Our findings may have limited longevity as model capabilities change frequently with new versions, performance benchmarks shift with architectural improvements, and entirely new models emerge that could surpass current leaders. The accelerated pace of development means our performance rankings may become outdated relatively quickly, and the evaluated models themselves may be updated in ways that alter their capabilities.

This dynamic landscape necessitates ongoing longitudinal evaluation to track performance consistency and monitor improvements over time. Future research should establish standardized benchmarking protocols that can be consistently applied across different model generations, enabling meaningful trend analysis in medical dialogue synthesis capabilities. The temporal limitation underscores the need for continuous re-evaluation as the field advances, ensuring assessments of LLM performance in healthcare applications remain current and relevant.

### 4.6. Implications for Practice, Education, and Research

The high quality of these LLM-generated synthetic dialogues supports their utility in medical education, particularly for training communication skills, facilitating case-based discussions, and preparing trainees for diverse patient interactions in plastic surgery. Integrating LLMs into Virtual Standardized Patients (VSPs) can provide a safe, accessible, and scalable solution for simulation-based learning. The strong descriptive performance in empathy is valuable, as empathy is both critical and challenging to teach. LLM scenarios can be tailored for training or evaluating empathetic responses in sensitive or challenging situations not easily replicated with human standardized patients. This is useful for teaching bedside manners, cultural sensitivity, delivering difficult news, or discussing aesthetic surgery expectations and psychological well-being. Models maintaining consistent personas and empathy are well-suited for preparing clinicians for remote care and could offer scalable training for telemedicine consultation skills.

Beyond traditional text-based simulations, LLMs have the potential to animate virtual reality (VR) avatars, both patients and physicians by generating dynamic, naturalistic dialogue and emotionally nuanced interactions [50]. This capability can dramatically enhance the realism of VR-based medical training by giving virtual characters coherent personas, medical reasoning, and empathetic responses [51]. Ambient sensing technologies, such as wearable devices, biosensors, and room-based acoustic sensors, increasingly capture physiological and behavioral data in real time. When LLMs are combined with sensor technologies such as gaze tracking, facial expression analysis, haptic sensing, and physiological monitoring, these avatars become not only verbally articulate but also responsive to non-verbal cues [52]. This multimodal integration enables virtual agents to adapt in real time based on trainee behavior or patient emotion, significantly increasing simulation fidelity and the educational value of immersive VR environments [53].

For research, these LLMs can generate synthetic clinical conversational data for developing and testing NLP models, pre-testing chatbots, and simulated adversarial testing. Our study provides a robust methodological framework and a seven-criterion rubric for evaluating such dialogues, addressing the multi-dimensional nature of clinical communication quality.

While this study did not evaluate LLMs for real-time clinical decision support or direct patient interaction, their ability to generate accurate, empathetic content suggests potential for assisting in drafting patient education materials or initial template responses, always under strict clinical oversight and validation. Based on this study, direct, unvalidated patient-facing use is not recommended, underscoring the need for rigorous, domain-specific validation before any high-stakes medical deployment.

The non-significant ANOVA results, alongside high descriptive scores, suggest that several leading LLMs have achieved comparable competence for generating high-quality clinical dialogues. Future differentiation might hinge on performance in more specialized or complex reasoning tasks, nuanced scenarios, or efficiency with less prompt engineering. Dialogue quality is an interplay of LLM capability, prompt specificity, and rubric sensitivity; non-significant findings could be influenced by any of these, with subtle differences perhaps only revealed by highly optimized prompts or more granular metrics.

### 4.7. Ethical Considerations

Demographic patterns in our synthetic dialogues, such as the complete absence of male patients and limited age representation (excluding pediatric or geriatric populations), raise significant ethical concerns about bias perpetuation. Such systematic biases, likely reflecting LLM training data, risk inadequately preparing healthcare providers for diverse patient populations, potentially contributing to care disparities and amplifying existing healthcare inequities [54].

While synthetic dialogues mitigate direct privacy risks from output, ethical considerations persist regarding input. Many commercial LLMs are not inherently HIPAA-compliant and might retain or analyze input prompts, creating vulnerabilities if real patient data were used in generation requests. Robust data governance is crucial for preventing the inadvertent inclusion of real patient data in these processes. Furthermore, the accessibility of high-quality synthetic medical dialogues means medical knowledge presentation is no longer exclusive to healthcare professionals. This raises concerns about patients encountering medical content of varying provenance online, necessitating physician oversight for the appropriate use and interpretation of any AI-generated medical content.

### 4.8. Future Directions

Our findings provide compelling evidence that LLM-generated synthetic dialogues represent not merely a technological advancement but a transformative tool that could fundamentally reshape medical education, clinical training, and healthcare AI research and development. Future research should aim to expand the scope of LLM evaluation by including a wider range of medical and surgical specialties and a larger, more diverse set of clinical scenarios, including those that are more complex or present unique ethical challenges. Incorporating a broader panel of evaluators, potentially including patients themselves to assess criteria like realism and empathy from their unique perspective, would add valuable dimensions to the assessment. Longitudinal studies are needed to assess the actual educational impact of integrating LLM-generated synthetic dialogues into medical training curricula.

Future comparative studies could benchmark LLM-generated dialogues not only against each other but also against dialogues scripted by human clinical experts or transcripts of anonymized and real patient interactions to provide a more definitive assessment of their quality relative to human standards. For future studies involving real patients, it will be possible to incorporate sensor-derived data (such as heart rate variability, facial expressions, and speech patterns) collected via wearables, microphones, or cameras. This would enable direct comparison between sensor-informed real interactions and LLM-generated synthetic dialogues. Notably, several of the synthetic transcripts in our study included visual and behavioral descriptors within parentheses (e.g., “[patient frowns]” or “[smiles gently]”), suggesting a latent capacity for simulating non-verbal cues. In practice, these cues could be generated or validated using computer vision tools and affective computing techniques, providing a rich multimodal dataset to evaluate how well LLMs simulate physiological responses and behavioral signals [55].

Sensor technology can be employed in trainees interacting with VSPs powered by LLMs, to assess cognitive and emotional engagement [56]. For instance, in virtual reality VSPs, eye-tracking sensors can capture visual attention and decision-making behavior, while voice analysis tools can evaluate speech dynamics, empathy, and hesitation [57]. These biosensors’ multimodal integration can be used to provide targeted feedback or adapt the VSP’s behavior in real time, thereby transforming static simulations into responsive, interactive learning environments. Similarly, when patients interact with LLM-driven synthetic physician avatars in VR or digital health environments, sensor and VR technologies, such as facial emotion recognition, gaze tracking, and physiological monitoring, can be used to simulate and respond to patient affect, thereby increasing the ecological validity and fidelity of the encounter [58]. This bi-directional sensing not only enhances realism but also supports research into patient experience, communication strategies, and affective AI.

Systematic investigation into the impact of different prompting strategies, such as zero-shot, few-shot learning, chain-of-thought variants [59], or detailed role-playing instructions on dialogue quality and specific attributes like empathy or accuracy, is warranted. The development and validation of objective, automated metrics for dialogue quality, which correlate with expert human judgment, would complement subjective ratings and improve the scalability and efficiency of future evaluations. 

Retrieval-Augmented Generation (RAG) offers a powerful dual role in this ecosystem [60]. First, RAG can enhance the factual grounding and coherence of synthetic transcripts during generation by incorporating verified medical knowledge in real time. It can further support bias mitigation by grounding outputs in diverse, curated datasets. Second, synthetic transcripts can serve as a foundation for prototyping and refining RAG-based frameworks that extract structured medical information from patient–physician conversational data, such as symptoms, diagnoses, or social determinants of health [61]. These frameworks can then be validated and deployed on real-world, de-identified transcripts to support clinical research, decision support, documentation automation, and patient-facing tools for recalling medical information that may have been missed during consultations. These conversations can be automated to serve as high-quality, privacy-preserving training data for building sophisticated ambient clinical intelligence (ACI) systems. These systems, once robustly trained on vast datasets of synthetically generated dialogues, can then operate in real clinical workflows by passively listening to actual patient–physician interactions. The AI leverages its learned patterns and medical knowledge from the synthetic data to automatically transcribe, analyze, and extract key clinical information, subsequently drafting comprehensive and structured medical notes. Furthermore, this training enables the AI to summarize lengthy conversations into concise, clinically relevant overviews; identify crucial diagnostic and treatment information; and even suggest appropriate medical codes (e.g., ICD-10, CPT), thereby streamlining the entire documentation process and significantly reducing the administrative burden on physicians. This automation allows clinicians to focus more directly on patient care while simultaneously improving note accuracy, completeness, and adherence to medical standards.

To address demographic bias, future work should incorporate prompt engineering that explicitly varies patient age, gender, ethnicity, and clinical conditions [62]. Name randomization and diversity constraints can reduce repetition. Including demographic parity checks and expanding evaluator diversity will further support equitable and representative synthetic dialogue generation.

Future research should investigate the development of domain-specific medical LLMs through fine-tuning on curated clinical datasets, which may achieve superior performance compared to general-purpose models in generating specialized medical dialogues. Future evaluations should also incorporate computational sentiment analysis to provide an objective assessment of emotional tone and affective appropriateness in synthetic medical dialogues, complementing expert clinical evaluation with standardized emotional analysis metrics. As new LLM architectures and agent-based systems emerge, their specific strengths and weaknesses for generating clinical dialogues should be evaluated. Finally, given the descriptive excellence observed, future work could explore if these LLMs maintain such high quality in more challenging scenarios requiring deeper reasoning or nuanced emotional intelligence.

## 5. Conclusions

This study provides robust evidence that LLMs such as ChatGPT 4.5, ChatGPT 4o, Claude 3.7 Sonnet, and Google Gemini Pro 2.5 can generate high-fidelity synthetic patient–physician conversations that are accurate, realistic, and usable. Across a diverse set of plastic surgery scenarios, all four models consistently achieved high ratings across seven core clinical communication dimensions when assessed by medically trained reviewers. While descriptive differences revealed subtle strengths, particularly in empathy, realism, and medical completeness, the absence of statistically significant variation among models suggests that state-of-the-art LLMs have reached a high baseline of competence in this domain.

These findings underscore the potential of LLM-generated synthetic dialogues to serve as a valuable resource for medical education, healthcare simulation, and AI development. By offering scalable, privacy-preserving alternatives to real patient conversations, synthetic dialogues open new avenues for training, evaluation, and research, particularly in settings where data access is restricted. However, this promise must be tempered by awareness of current limitations, including demographic bias, verbosity, and lack of diversity in generated content. These challenges highlight the need for continued oversight, thoughtful prompt engineering, and inclusive design practices.

As the capabilities of LLMs evolve, so too must our frameworks for evaluating their outputs. Future efforts should focus on expanding to additional specialties, diversifying scenario complexity, incorporating patient perspectives, and benchmarking against real clinical interactions. Importantly, synthetic data generation must remain grounded in ethical responsibility, ensuring that inclusivity, transparency, and clinical validation remain central to development. With careful stewardship, LLM-generated dialogues can begin to complement existing tools in simulation-based medical education and research.

## Figures and Tables

**Table 1:** Mean (SD) Likert Scale Scores for Each Evaluation Criterion by LLM Model.

Evaluation Criterion	Likert (Mean ± SD)			
	ChatGPT 4.5	ChatGPT 4o	Claude 3.7 Sonnet	Google Gemini Pro 2.5
Medical Accuracy	4.96 ± 0.18	4.73 ± 0.45	4.9 ± 0.3	5.00 ± 0.00
Realism	4.93 ± 0.25	4.56 ± 0.56	4.83 ± 0.38	5.00 ± 0.00
Persona Consistency	4.9 ± 0.30	4.67 ± 0.54	5.00 ± 0.00	5.00 ± 0.00
Fidelity to Prompt	4.7 ± 0.79	4.73 ± 0.45	4.96 ± 0.18	4.9 ± 0.3
Empathy	4.93 ± 0.25	4.5 ± 0.73	4.96 ± 0.18	4.80 ± 0.4
Relevancy	5.00 ± 0.00	4.73 ± 0.45	5.00 ± 0.00	5.00 ± 0.00
Usability	4.96 ± 0.18	4.76 ± 0.43	4.93 ± 0.25	5.00 ± 0.00

Note: Scores are on a 5-point Likert scale (1 = Very Poor, 5 = Excellent). SD = Standard Deviation.

**Table 2:** Linguistic Features of LLM-Generated Dialogues.

LLM	ChatGPT4.5	ChatGPT4o	Claude 3.7 Sonnet	Gemini 2.5 Pro
Word Count	860.3	533.8	1889.0	3688.3
Turn Count	48.5	27.8	101.3	97.7
Physician Words	486.7	335.5	1067.0	2339.7
Patient Words	373.6	198.3	822.0	1348.7
Physician-to-Patient Ratio	1.3	1.7	1.3	1.7
Lexical Diversity	0.5	0.6	0.4	0.3
Flesch Reading Ease	65.2	54.2	64.6	63.7
FK Grade Level	6.6	7.8	6.4	7.2
Clinical Terms	38.6	27.9	79.9	160.4
Filler Words	134.3	87.0	277.2	565.1
Empathic Phrases	5.3	2.9	8.2	12.6

## Data Availability

The original contributions presented in this study are included in the article/Supplementary Material. Further inquiries can be directed to the corresponding author.

## Supplementary Materials

The following supporting information can be downloaded at: https://www.mdpi.com/article/10.3390/s25144305/s1, Table S1: Rubric for grading.
