# Supplementary material for: Synthetic Patient–Physician Conversations Simulated by Large Language Models: A Multi-Dimensional Evaluation
Source: Sensors (Basel). 2025 Jul 10;25(14):4305. doi: 10.3390/s25144305 (PMC12298718; doi:10.3390/s25144305)
Supplement: Supplementary file 1 [file sensors-25-04305-s001.zip › sensors-3714223-supplementary.pdf]

Supplementary Table S1: Rubric for grading

Metric	Question for Evaluator	1 - Very Poor	2 - Poor	3 - Adequate	4 - Good	5 - Excellent
**1. Realism**	How well does the transcript emulate a genuine, natural conversation between the defined roles (e.g., patient and physician)?	Highly artificial; language is very stilted, unnatural flow, lacks any believable human interaction.	Mostly artificial; significant issues with natural language or flow, limited believability.	Mixed; some aspects feel natural, while others are artificial. Basic believability.	Mostly natural and believable; language flows well with only minor artificiality.	Very natural and highly believable; language flows exceptionally well, convincingly mirrors human conversation.
**2. Persona Consistency**	How well did the LLM maintain consistent and believable characters/personas for both the patient and the physician throughout the entire transcript (and across linked pre-op/post-op	Personas are highly inconsistent, contradictory, or completely unbelievable; frequent out-of-character moments.	Personas show noticeable inconsistencies or lack believability at several points.	Personas are generally consistent, with only minor lapses or a lack of depth; basically believable.	Personas are consistently maintained and largely believable with distinct characteristics.	Personas are exceptionally consistent, well-developed, nuanced, and highly believable throughout.

	transcripts, if applicable)?					
**3. Fidelity (to the Prompt)**	How accurately and faithfully does the transcript adhere to the specific details and constraints of the prompt/scenari o it was asked to generate?	Gross deviations from the prompt; most key instructions or scenario details are missed or incorrect.	Significant deviations from the prompt; several key instructions or scenario details are missed/incorr ect.	Generally aligns with the prompt but with noticeable deviations or omissions in some areas.	Mostly aligns accurately with the prompt; only minor or insignificant deviations or omissions.	Accurately and thoroughly reflects the prompt; all key instructions/scenar io details are precisely represented.
**4. Medical Accuracy**	How accurate is the medical information presented in the transcript, including terminology, discussion of conditions, treatments, and advice?	Contains significant, potentially harmful medical inaccuracies or gross misinformation .	Contains multiple noticeable medical inaccuracies, some of which could be misleading or are clearly incorrect.	Generally medically sound, but may contain minor or isolated inaccuracies, or overly simplistic information that isn't harmful.	Medically accurate with very few, if any, trivial inaccuracie s; terminology is used correctly and appropriatel y.	Consistently high level of medical accuracy throughout; information is correct, precise, current, and appropriately detailed.

**5. Empathy**	To what extent does the physician (and the overall physician-patient interaction) demonstrate empathy, understanding, and validation of the patient's perspective, concerns, and emotions?	Physician appears dismissive, cold, or completely lacks empathetic responses; patient concerns are ignored/invalidated.	Minimal or superficial empathetic responses; physician shows little understanding or validation of patient's feelings.	Some attempts at empathy are present (e.g., acknowledgement of feelings), but may feel formulaic or inconsistent.	Physician consistently demonstrates understanding, validation, and appropriate empathetic responses to patient cues.	Physician skillfully and genuinely conveys deep understanding, compassion, and validation; empathetic communication is well-integrated.
**6. Relevancy**	How pertinent and appropriate is the content of the transcript to the specified topic, procedure, or consultation type described in the	Content is almost entirely off-topic, inappropriate, or addresses completely irrelevant concerns.	Content has significant portions that are off-topic, inappropriate, or address irrelevant concerns.	Content is largely relevant and appropriate, but with some noticeable tangential or mildly irrelevant elements.	Content is mostly relevant, focused, and appropriate, with only minor or insignificant tangential elements.	Content is highly relevant, sharply focused, and directly/comprehensively addresses the scenario's core aspects.

	prompt/scenario?					
**7. Usability**	How suitable and effective is this transcript for its intended purpose (e.g., for training, research, demonstration, or a specific analytical task)?	Not at all useful for the intended purpose; fundamentally unclear, confusing, incomplete, or unusable.	Limited usability; significant issues with clarity, completeness, or applicability for the purpose.	Moderately useful; generally understandable/applicable but with clear limitations or requiring effort to use.	Quite useful; clear, mostly complete, and largely effective for the intended purpose with minor limitations.	Highly useful and very effective; exceptionally clear, well-structured, and perfectly suited for the purpose.
